# A Case of Nemaline Myopathy With Sleep-Related Hypoventilation Diagnosed Using Polysomnography During Daytime Napping

**DOI:** 10.7759/cureus.52907

**Published:** 2024-01-25

**Authors:** Kanako Tamura, Kiyohide Komuta, Keijirou Yamauchi, Masashi Yokoyama, Hiroshi Morishita

**Affiliations:** 1 Department of Respiratory Medicine, Osaka Prefectural Hospital Organization, Osaka Habikino Medical Center, Osaka, JPN

**Keywords:** pseudo-central event, transcutaneous pco2, nemaline myopathy, polysomnography, sleep related hypoventilation

## Abstract

This is the case of a 49-year-old woman who was admitted to the hospital for a close examination of pulmonary hypertension; however, the next morning, she developed carbon dioxide (CO_2_) narcosis and was started on artificial ventilation. As pulmonary arterial hypertension was ruled out, the patient was extubated, and 24-hour transcutaneous partial pressure of carbon dioxide (PCO_2_)(transcutaneous carbon dioxide (TcPCO_2_)) monitoring was performed to diagnose sleep-related hypoventilation. Polysomnography (PSG) during daytime napping revealed markedly decreased chest motion and a “pseudo-central event," which was neither central nor obstructive hypopnea. Based on the PSG results and physical examination findings, a neuromuscular disorder was suspected, and a muscle biopsy was performed to diagnose nemaline myopathy. Neuromuscular diseases are widely recognized for their association with sleep-disordered breathing; thus, sleep-related hypoventilation should also be considered. Monitoring of TcPCO_2_ and PSG are useful tools in identifying the cause of hypoventilation; however, overnight PSG may cause CO_2_ narcosis in some diseases. In such cases, PSG may be beneficial during daytime napping.

## Introduction

In the International Classification of Sleep Disorders, Third Edition, sleep-related breathing disorders are classified into five groups. Among them, there is the sleep-related hypoventilation disorder group, further divided into six conditions namely, obesity hypoventilation syndrome, congenital central alveolar hypoventilation syndrome, late-onset central hypoventilation with hypothalamic dysfunction, idiopathic central alveolar hypoventilation, sleep-related hypoventilation due to a medication or substance, and sleep-related hypoventilation due to a medical disorder [1]. The American Academy of Sleep Medicine suggested the following criteria for sleep-related hypoventilation in adults: the arterial partial pressure of carbon dioxide (PCO_2_) (or surrogate) during sleep increases >55 mmHg for ≥10 minutes, or the arterial PCO_2_ (or surrogate) during sleep increases >10 mmHg compared with awake supine values exceeding 50 mmHg for ≥10 minutes [2]. This case was finally diagnosed as nemaline myopathy and fell into the category of sleep-related hypoventilation due to a medical disorder. In this report, we focus on the diagnostic process of determining the cause of hypoventilation.

## Case presentation

**Figure 1:**
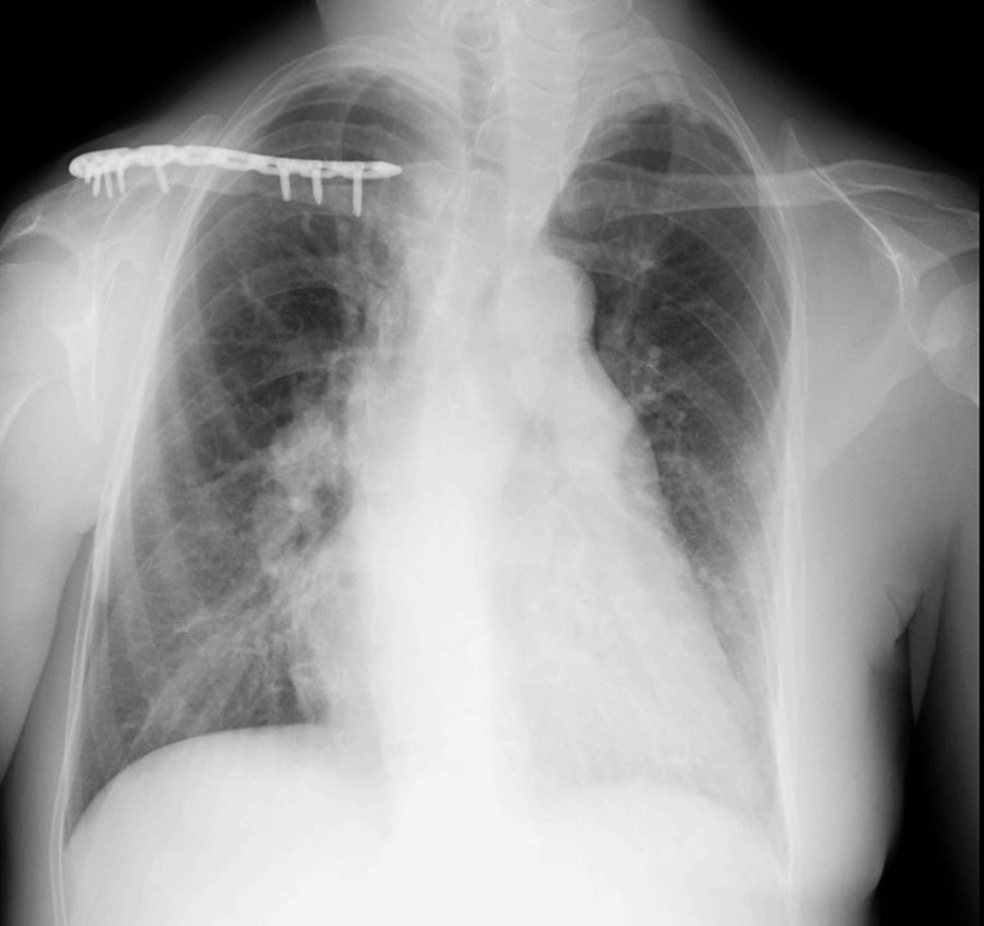
The patient's chest radiograph obtained on admission

The patient, a 49-year-old woman, had been aware of mild breathlessness on exertion since she was 20 years old, had leg edema since the age of 38, and had hypertension since the age of 48, both of which were treated with antihypertensive drugs and diuretics. At the age of 47, while dozing in a chair, she fell off the chair and fractured her collarbone. She had no noteworthy family history, and she had never smoked. She worked in an office. She was referred to the Department of Cardiology at our hospital for the evaluation of increased leg edema and hypoxia. At the time of the initial examination, she presented with facial edema and mucocutaneous cyanosis in addition to leg edema, and her peripheral capillary oxygen saturation (SpO_2_) was 90%. Respiratory sounds were normal, and no sputum or cough were noted. Chest radiography displayed cardiac enlargement (Figure 1), and echocardiography displayed preserved contractility with no evidence of valvular disease.

**Figure 2:**
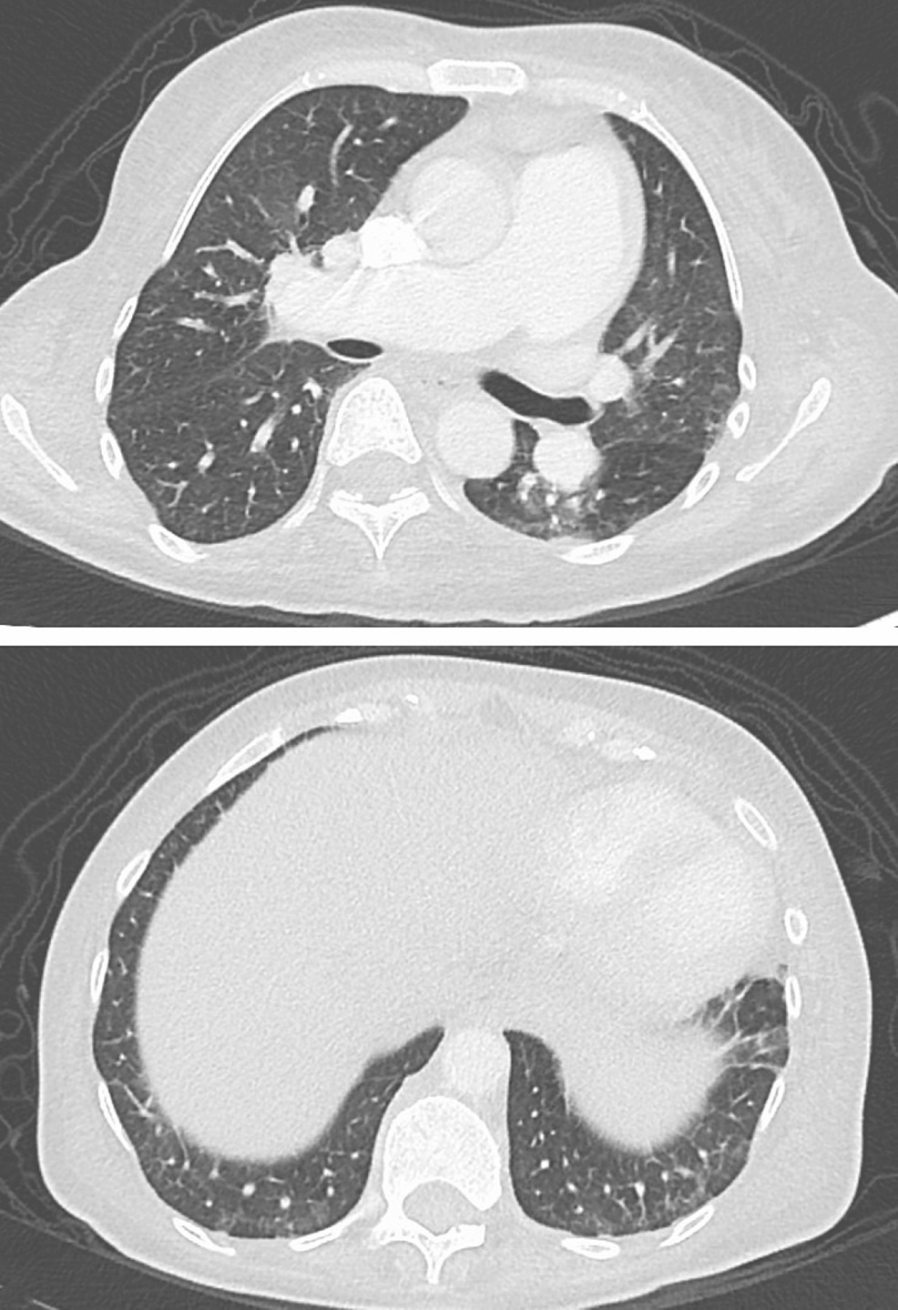
The patient's chest computerized tomography obtained on admission

However, her tricuspid regurgitation pressure gradient (TRPG) had increased to 55 mmHg. A CT scan of the chest showed no abnormality in the lung field and pulmonary artery enlargement (Figure 2), but no thrombus was found in the pulmonary artery on chest contrast-enhanced CT; therefore, pulmonary arterial hypertension was suspected, and the patient was admitted to the hospital.

The patient was treated with an additional diuretic and 1 L/min of oxygen. The next morning, she was found to have altered mental status with a Glasgow Coma Scale (GCS) of five.

The blood gas values were as follows: acid-base balance (pH) of 7.04, the arterial PCO_2_ (partial pressure of arterial carbon dioxide (PaCO_2_)) of 170 mmHg, the arterial partial pressure of oxygen (PO_2_) (partial pressure of oxygen (PaO_2_)) of 100 mmHg in O_2_ 3L/min, and the alveolar to arterial oxygen pressure difference was -77 mmHg, indicating respiratory acidosis. Treatment was started with noninvasive positive pressure ventilation (NPPV), but her mental status did not improve; therefore, artificial ventilation was started.

The cause of hypoventilation was unknown, but the cardiologist in charge of her thought the loss of consciousness was due to pulmonary arterial hypertension. Therefore, she was transferred to a specialized cardiology hospital with a pulmonary circulation department while on a ventilator. At the hospital where she was transferred, her tricuspid regurgitation pressure gradient (TRPG) had decreased to 15 mmHg, and it was concluded that she did not suffer from pulmonary arterial hypertension. She remained intubated and was readmitted to our hospital the next day, where respiratory physicians were assigned to her.

The patient weighed 56 kg with a body mass index of 21 and a GCS score of E4VTM6 (on ventilatory management). Moreover, edema was observed on her face and in both lower legs. Chest radiography revealed cardiomegaly and scoliosis, with a Cobb angle of 28 °(Figures 1-2).

No blood gas analysis was performed before the onset of CO_2_ narcosis, and post-intubation blood gases revealed values within the pH of 7.5 and PaCO_2 _of 40 mmHg ranges.

**Figure 3:**
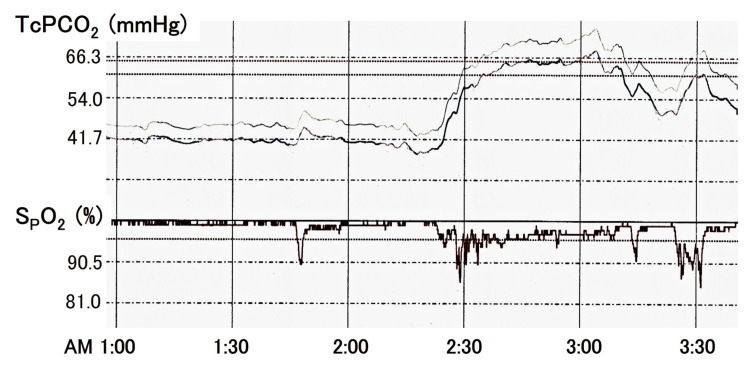
Transcutaneous carbon dioxide (TcPCO2) monitoring during non-invasive positive pressure ventilation (IPAP/EPAP: 15/5 cmH2O, FiO2: 30%, RR: 20 cycles/min) The upper part of the TcPCO_2_ curve is the actual value and the lower part is the corrected difference from PaCO_2_. IPAP: inspiratory positive airway pressure; EPAP: expiratory positive airway pressure; cmH_2_O: centimeter of water;  FiO_2_: fraction of inspired oxygen; RR: respiratory rate; PaCO_2_: artial pressure of carbon dioxide

Sleep-related hypoventilation was suspected because she had no abnormalities in her lung field or pulmonary blood vessels and developed CO_2_ narcosis after sleep; furthermore, according to her husband, she has frequently dozed off during the day for the past two years. Her mental condition had improved with artificial ventilation, and if the hypoventilation was sleep-related, some ventilation was expected to be maintained upon awakening; therefore, the patient was extubated. After extubating, it was revealed that she had been aware of daytime sleepiness since two years ago. Non-invasive positive pressure ventilation was initiated for 24 hours, and SpO_2_ and transcutaneous carbon dioxide (TcPCO_2_) were monitored. Anticipating severe hypoventilation for several days after extubating, the staff checked on her to see if the TcPCO_2_ was above 70 mmHg to assess her level of consciousness, even at night. The TcPCO_2_ monitoring on the fifth day after extubating is displayed in Figure 3.

Under NPPV (S/T mode, inspiratory positive airway pressure (IPAP)/expiratory positive airway pressure (EPAP): 15/5 centimeters of water (cmH_^2^_O), fraction of inspired O_2_: 20%-30%, respiratory rate (RR): 20 cycles/min), TcPCO_2_ remained in the 40-mmHg range upon awakening but increased to 60-70 mmHg upon falling asleep, accompanied by hypoxia.

The patient's spontaneous breathing was barely detectable during sleep, and forced ventilation was triggered. Suspecting obstructive sleep apnea (OSA), we raised the EPAP during sleep from 4 cmH_2_O to 8 cmH_2_O, which only slightly improved oxygenation and did not increase spontaneous breathing. Therefore, we concluded that obstructive apnea is not the only cause of hypoventilation, although obstructive apnea may be present during sleep.

With the increase in TcPCO_2_ during sleep, a diagnosis of sleep-related hypoventilation was established; however, the cause was unknown. Obesity hypoventilation, hypothalamic dysfunction, and drug-induced hypoventilation were excluded as causes of sleep-related hypoventilation. Therefore, the cause was narrowed down to alveolar hypoventilation due to a medical disorder or late-onset congenital central alveolar hypoventilation (CCHS). The patient had scoliosis with a Cobb angle of 28°; however, the thoracic deformity was mild and not considered a cause of severe hypoventilation. Based on these findings, we strongly suspected a late-onset form of CCHS.

The patient was able to wean off NPPV when awake but continued to utilize it at night. We wanted to perform a nighttime polysomnography (PSG) for close examination of sleep disturbance but decided that if the patient had CCHS, an overnight PSG without NPPV would put the patient at risk of developing CO_2_ narcosis. Therefore, the attending physician observed the patient at the bedside and performed PSG during daytime napping without NPPV.

**Figure 4:**
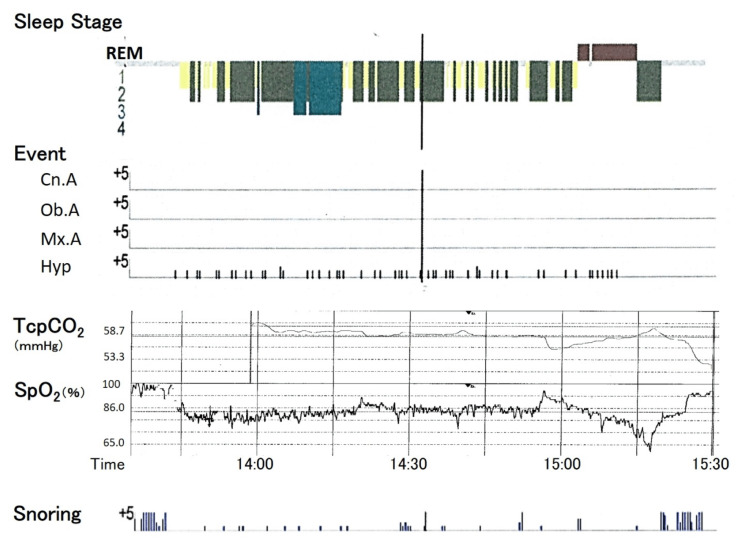
Polysomnography performed in two hours during daytime napping REM: rapid eye movement; Cn.A: central apnea; Ob.A: obstructive apnea; Mx.A: mixed apnea; Hyp: hypopnea; TcPCO_2_: transcutaneous carbon dioxide; SpO_2_: oxygen saturation

**Figure 5:**
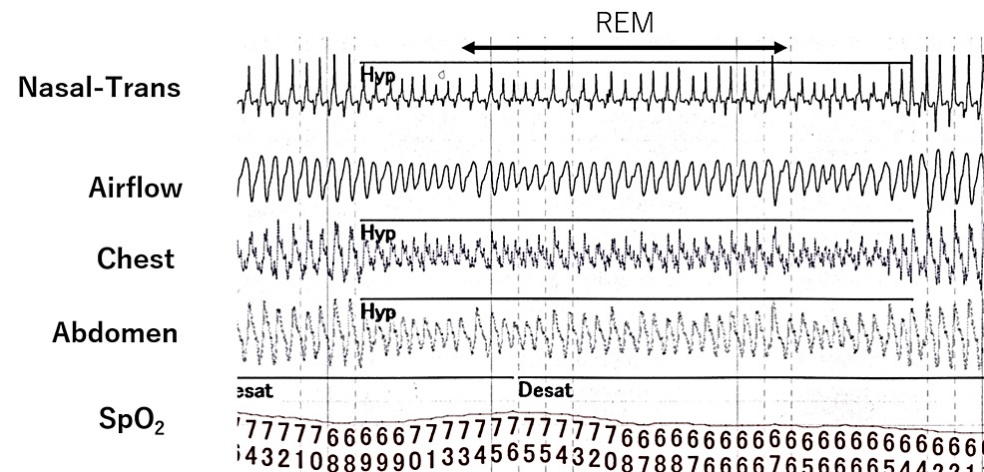
Electromyograph recording during polysomnography The waveform of chest motion is smaller than that of the abdomen in rapid eye movement (REM) sleep. This waveform can be considered a “pseudo-central event.” Hyp: hypopnea; Desat: desaturate; SpO_2_: oxygen saturation

During naps, the PSG revealed chest movements that were significantly attenuated compared to abdominal movements, although post-onset snoring was slight. The examination was terminated when SpO_2_ dropped to 60% two hours after the start of the examination. Her blood gas levels before the examination were as follows: pH of 7.33, PaCO_2_ of 53 mmHg; however, immediately after the examination, her blood gas levels were pH: 7.28, PaCO_2^:^ _68 mmHg. Fortunately, the patient was conscious. The results of the two-hour PSG demonstrated an apnea/hypopnea index of 35/h, all of which were hypopneas (Figures 4-5).

Hypopnea was defined as central hypopnea, according to the guidelines [3]. However, it could not be determined whether this indicated the presence of CCHS. The patient's chest movements were significantly weakened during PSG, prompting suspicion of neuromuscular disease. Therefore, we considered ruling out neuromuscular disease necessary before proceeding with the diagnosis of CCHS. Upon commencing rehabilitation, we discovered that the patient's neck was drooping, she had an agitated gait, and her limb muscle strength had decreased. The patient's developmental history revealed that her neck control developed slowly, and she started walking several months later than the average during infancy. Results of the pulmonary function test displayed a restrictive pattern with a forced vital capacity of 61% and a forced expiratory volume in one second to a forced vital capacity ratio of 89.8%. Furthermore, the six-minute walk distance was 320 m, which was quite short even after taking age into account (Table 1).

**Table 1:** Results of pulmonary function and exercise capacity FVC: forced vital capacity; FEV1 : forced expiratory volume; TLC : total lung capacity; DLCO: carbon monoxide diffusing capacity; VA: alveolar volume; 6MWD: six-minute walk distance

Parameters	Results
FVC (L)	1.78
FEV1(L)	1.6
FVC (%predicted)	61.0
FEV1/FVC (%)	89.8
TLC (L)	3.43
DLCO (ml/min/mmHg)	14.5
DLCO/VA (ml/min/mmHg/L)	5.26
6MWD (m)	320

Based on these findings, we suspected sleep-related hypoventilation due to neuromuscular diseases, particularly congenital myopathy. Blood gases during daytime awakening without NPPV were pH of 7.3-7.4 and PaCO_2_ of 50-55 mmHg, with type 2 respiratory failure even when awake, but pH was in the normal range when awake. She was treated with NPPV (S/T mode, IPAP/EPAP: 16/8.4 cmH2O, RR: 15 cycles/min, no oxygen) only while sleeping. After discharge from the hospital, she was referred to the Department of Neurology at Nara Medical University, Kashihara, Japan, where a diagnosis of nemaline myopathy was made based on a biopsy of the biceps brachii muscle. She continues to receive NPPV treatment only during sleep without recurrence of loss of consciousness, but her general muscle weakness has worsened.

## Discussion

Nemaline myopathy is the most common form of congenital myopathy, and currently, 12 types of genetic abnormalities have been identified [4]. There is still no fundamental treatment for this disease, which has a wide range of severity. There are reports of cases of nemaline myopathy diagnosed in adulthood due to respiratory failure and pulmonary hypertension [5]. This patient also had pulmonary hypertension on admission. It is widely known that hypoxia causes pulmonary arteriolar vasospasm, which increases pulmonary arterial pressure. Since the pulmonary hypertension in this case was quickly improved by intubation and ventilation, we considered that persistent hypoxemia due to sleep-disordered breathing caused pulmonary arteriolar spasm.

Central alveolar hypoventilation is a representative disease of sleep-related hypoventilation and a genetic disorder that presents with an impaired ventilatory response. Mutations of the PHOX2B gene are found in most of the patients with CCHS [6]. We have late-onset CCHS patients, and we consider it a disease that should be kept in mind when hypoventilation of an unknown cause occurs.

On the other hand, patients with neuromuscular diseases have a different mechanism of sleep-related hypoventilation from that of CCHS. In general, diaphragm movements are more involved in ventilation during REM than intercostal muscles and other auxiliary respiratory muscle activities [7]. Normal patients can maintain ventilation volume only by diaphragm movements even if other respiratory muscle activities are decreased, but patients with neuromuscular diseases with diaphragm weakness cannot compensate for ventilation volume only by diaphragm movements [8]. In relation to this, a peculiar type of hypopnea called a “pseudo-central event” has been reported during the REM period in patients with neuromuscular diseases [9]. This type of hypopnea is neither obstructive nor central and is said to be caused by a decrease in chest movement during sleep compared to that in the abdomen due to a decrease in diaphragm muscle strength. The hypopnea in this case showed the same characteristics, and it is possible that a "pseudo-central event" was observed (Figure 5). Although we did not assess diaphragmatic function in this report, diaphragmatic echocardiography can be performed noninvasively and should be performed when treating such cases in the future.

Finally, we would like to discuss why the patient developed CO_2_ narcosis after hospitalization. There were two possible causes of her daytime hypercapnia: one was due to restrictive lung function, and the other was due to hypercapnia from sleep-related hypoventilation that carried over into the daytime. She has always had hypercapnia, and her ventilatory response to CO_2_ would be expected to have been reduced. Therefore, we believe that the administration of oxygen after hospitalization induced CO_2_ narcosis. Other cases of CO_2_ narcosis induced by administering oxygen to patients with neuromuscular diseases have been reported [10]. We must first check for ventilation when treating a hypoxic patient, even if we do not suspect respiratory disease.

## Conclusions

In the practice of sleep-related hypoventilation, it is essential to exclude hypoventilation due to obstructive or restrictive ventilation disorders of the lungs, such as chronic obstructive pulmonary disease (COPD), interstitial pneumonia, and thoracic restrictive disease. In this case, we ruled out respiratory disease based on breath sounds, chest imaging, and respiratory function tests.

Since respiratory disease could be ruled out, the cause of sleep-related hypoventilation was assumed to be late-onset congenital central hypoventilation syndrome, neuromuscular disease, or other medical conditions. We suspected neuromuscular disease due to the “pseudo-central event” on PSG during daytime napping and restrictive patterns in the pulmonary function test.

Sleep-related breathing disorders are well-known to coexist with neuromuscular disorders, whereas sleep-related hypoventilation may be less well-recognized. While TcPCO_2_ and PSG are very helpful in determining the cause of hypoventilation, some patients who undergo PSG overnight run the risk of experiencing CO_2_ narcosis. In such cases, PSG in a safe environment, even for a short time during daytime napping, may be useful for diagnosis.
